# Characterization of non-O157 enterohemorrhagic *Escherichia coli* isolated from different sources in Egypt

**DOI:** 10.1186/s12866-024-03636-3

**Published:** 2024-11-21

**Authors:** Omnia T. Bahgat, Dina E. Rizk, Hany I. Kenawy, Rasha Barwa

**Affiliations:** Microbiology and Immunology Department, Faculty of Pharmacy, Mansoura University, Mansoura, 35516 Egypt

**Keywords:** Non O157 EHEC- ERIC, Biofilm, Serum resistance, Shiga toxin, Intimin

## Abstract

**Background:**

Enterohemorrhagic *Escherichia coli* (EHEC) O157 is implicated in serious food and water-borne diseases as hemorrhagic colitis (HC), and the potentially fatal hemolytic uremic syndrome (HUS). However, new players of non-O157 EHEC have been implicated in serious infections worldwide. This work aims at analyzing serotype and genotypic-based virulence profile of EHEC local isolates.

**Methods:**

A total of 335 samples were collected from different sources in Egypt. *E. coli* was isolated and subjected to serotyping. Non-O157 EHEC isolates were tested for virulence genes using PCR, phenotypic examination, phylogenetic typing, and molecular investigation by ERIC typing and MLST to disclose genetic relatedness of isolates. A heat map was used to identify potential associations between the origin of the isolates, their phenotypic and genotypic characteristics.

**Results:**

A total of 105 out of 335 isolates were identified as *E. coli*. Surprisingly, 49.5% of these isolates were EHEC, where O111, O91, O26 and O55 were the most prevalent serotypes including 38.46% from stool, 21.15% urine, 23.1% cheese, 9.62% meat products, 3.85% from both yogurt and sewage water. Screening 15 different virulence genes revealed that *sheA, stx2* and *eae* were the most prevalent with abundance rates of 85%, 75% and 36%, respectively. Fifteen profiles of virulence gene association were identified, where the most abundant one was *stx2*/*sheA* (19%) followed by *stx2/stx2g/sheA/eae* (11.5%). Both *stx2/sheA/eae* and *stx2/stx2g/sheA* were equally distributed in 9.6% of total isolates. Phylogenetic typing revealed that pathogenic phylogroups B2 and D were detected among clinical isolates only. Forty-six different patterns were detected by ERIC genotyping. MLST resolved three sequence types of ST70, ST120 and ST394. The heat map showed that 21 isolates were of 70% similarity, 9 groups were of 100% clonality.

**Conclusions:**

The prevalence of non-O157 EHEC pathotype was marginally higher among the food isolates compared to the clinical ones. The endemic ST120 was detected in cheese, necessitating crucial measures to prevent the spread of this clone. Clinical EHEC isolates exhibited a higher score, and combination of virulence genes compared to food and sewage water isolates, thereby posing a significant public health concern.

**Supplementary Information:**

The online version contains supplementary material available at 10.1186/s12866-024-03636-3.

## Introduction

Most *E. coli* strains are harmless [1]. By acquiring mobile genetic elements, through horizontal gene transfer, normally harmless *E. coli* can rapidly transform into a pathogen with a high adaptive capacity. Besides the prominent role of *E. coli* to be a contributor to intestinal diarrheal diseases, the majority of pathogenic isolates also result in extra-intestinal illnesses which are known as extraintestinal pathogenic *E. coli* (ExPEC) [2]. They can also lead to more than two million annual fatalities [3]. Feces and wastewater treatment of plants can both release infectious *E. coli* into the environment [4–6]. Raw milk and cheese can be a significant source of hazardous pathogenic *E. coli* to people [7]. There are nine different pathotypes of Diarrheagenic *E. coli* strains, including enterohemorrhagic *E. coli* (EHEC), enterotoxigenic *E. coli* (ETEC), enteropathogenic *E. coli* (EPEC), enteroinvasive *E. coli* (EIEC), enteroaggregative *E. coli* (EAEC), diffusely-adhering *E. coli* (DAEC), Shiga toxin-producing *E. coli* (STEC), adherent-invasive *E. coli* (AIEC), and cell-detaching *E.coli* (CDEC) [8]. The STEC are pathogenic bacteria that infect people through contaminated food and water. Shiga toxins (stx) are produced during STEC infections. Infection outcomes are determined by an array of strain and host variable and symptoms can range from mild to severe bloody diarrhoea, with HUS being a possible consequence. The EHEC is a prominent category of STEC that can produce either one or more stx which make up the main virulence characteristic of this pathogroup of *E. coli*. This pathotype causes a variety of infections, from barely detectable diarrhea to more serious manifestations such as the HC and the emergence of the potentially fatal HUS. EHEC infections are the leading factor of children’s acute renal failure in many countries, making kids and infants the most vulnerable patients [9, 10]. Whereas not all STEC induce HC or HUS, EHEC is generally reserved for those that do [11].

The serotype O157:H7 is regarded as the prototype of this pathogenic group as it was the initial cause the HC and HUS cases in the 80 s and since then it has become a contributor to many outbreaks across the globe [12]. While O157:H7 is the most prominent EHEC serotype, non-O157 EHEC strains are now recognized as hazardous causes of disease. O26, O103, O111, and O145 are the most prevalent non-O157 O-serogroups in Germany, accounting for one-third of EHEC outbreaks including severe diarrhea and life-threatening HUS [13]. These serogroups' detection rate has increased worldwide [14]. This increase may have been caused by increased awareness among labs or the creation of improved detection procedures, however, it may have been a result of the greater prevalence of infections with these pathogens that are linked to human and animal disease [15–17].

Toxin production, the formation of biofilm, iron acquisition systems, serum resistance ability, capsules, and adhesins are just a few of the many virulence factors that *E. coli* possesses [18]. These factors encourage tissue colonization, cause damage, and promote the spread of diseases. Due to these virulence traits, the microorganism can colonize anatomical environments, override host defense mechanisms, and start causing an inflammatory response in the host [18]. Shiga toxin 1 and 2, intimin, and enterohemolysin play vital roles in the O157 EHEC pathogenic mechanism [19–21].

The genus *Escherichia* is represented by a wide range of species including *E. coli*, *E. albertii*, *E. fergusonii*, and five cryptic *Escherichia* clades (I–V) [22]. Because of the nucleotide identity identified between clade I and *E. coli* strains, Escherichia clade I should be regarded as a subspecies of *E. coli* [23]. Based on the most recent phylogenetic assortment, *E. coli* strains have been assembled into eight phylogenetic classes that are A, B1, B2, C, D, E, F, and clade I [6]. Previous studies have shown that nonpathogenic strains belonged to A and B1 phylogenetic groups, meanwhile pathogenic ones belonged to B2 and D phylogenetic groups [24, 25] and [26]. As a result, phylogenetic clustering of *E. coli* strains is useful for displaying the relationship between phylotypes and diseases induced by the organism.

Determining the source of infection and the specific types of pathogens using various approaches such as molecular typing is a necessity to investigate the prevalence of hospitalized infections [27]. Moreover, accurate and quick molecular detection techniques are required to recognize pathogenic *E. coli* in food and animals in order to improve food safety and human health, as well as to minimize geographical extent of outbreaks [28]. Enterobacterial repetitive intergenic consensus polymerase chain reaction (ERIC-PCR) technique serves as DNA fingerprinting tool for assessing bacterial clonal variability [29]. As an outcome, ERIC-PCR was applied to examine the genetic similarity of EHEC isolates obtained from diverse sources. This also allows for the evaluation of the variable possible contamination sources [30].

Traditional molecular typing techniques such as phage typing, antibiotic resistance patterns analysis and plasmid profiling might be labor intensive, complex, difficult to distinguish between strains with high genetic similarity and are poorly transportable because they index variation that is hard to compare across laboratories. As a result, in epidemiological investigations, these approaches are not ideal for determining the source of infection [31]. To solve these issues, sequence-based typing methods have become the gold standard for epidemiological monitoring to study microbial population genetics. Multi-locus Sequence Typing (MLST) is a typing method that is widely used for clinically relevant bacterial species, and online databases have been developed, allowing data to be analyzed simply [32]. The MLST technique generates an allelic profile by analyzing the sequences of seven housekeeping genes. The produced allelic profile is summarized by the assignment of a sequence type in an electronic database. The closely related species are categorized into clonal complexes [33]. Bearing in mind the possibility of transmission of pathogenic *E. coli* to humans through consumption of contaminated food, this study aims to detect the prevalence of non-O157 EHEC isolates among different clinical, food and sewage sources in Egypt. In addition, monitoring of various virulence determinants is important to detect the potential of pathogenesis of these emerging strains.

## Material and methods

### Specimens’ collection

During the period from November 2018 to April 2020, a total of 118 clinical samples were collected, including 41 and 77 isolates from urine and stool, respectively and a total of 217 samples from different food sources including 93 animal product (meat, luncheon, smoked turkey, beef burger, ground beef and pastrami), 89 dairy products such as (cheese, yogurt and raw milk), 11 Fresh vegetables isolates (colored pepper, lemon and tomatoes), 15 chicken isolates (cooked and roasted), two fish isolates and seven sewage water isolates (supplementary Table 1).

Urine and stool samples were obtained from Mansoura university hospitals and private medical analysis laboratories from separate human cases after a written informed consent from the participant. The experimental protocol used in this research adheres to the ethical guidelines and principles of care, use, and handling of human subjects in medical research established by "The Research Ethics Committee, Faculty of Pharmacy, Mansoura University, Egypt" (code: 2022–126), which is governed by the World Medical Association's Code of Ethics (Declaration of Helsinki). Food samples (meat and dairy products) were obtained from one hundred eighty-two butchers’ shops and supermarkets, chicken isolates were obtained from fifteen poultry shops and fish isolates from two separate fish shop in Mansoura and Damietta cities, Egypt. Sewage isolates were obtained from sewage water from different places in Mansoura and Damietta cities. The fecal, meat, food samples were collected in sterile plastic containers and the milk, yogurt, sewage water and urine samples were collected in sterile falcons. Each individual sample was kept cool in icebox and transferred directly to the Microbiology and Immunology laboratory at the Faculty of Pharmacy, Mansoura university and stored at 4 °C till use; all samples were processed as fast as possible to avoid deterioration and contamination of samples.

### Isolation and identification of *E. coli*

The collected specimens were cultivated for isolation of *E. coli* in nutrient broth media that contain yeast extract (0.2%), peptone (0.5%), sodium chloride (0.5%) and incubated for 24 h at 37 °C. Then, appropriate inoculum subjected to subsequent isolation on MacConkey's agar selective media plates which were incubated at 37 °C for 24 h. All plates were then examined for colony morphology.

The suspected bacterial colonies that were identified by observation of lactose fermentation on MacConkey`s agar media and negative Gram staining were picked up and inoculated for further confirmation on Eosin methylene blue (EMB) agar plates [34]. Colonies with the characteristic green metallic sheen on EMB agar were subjected to additional confirmation using biochemical tests [35].

Food and sewage water samples were isolated according to Dickson et al. 1995 [36], where food samples (approximately 5 g) and liquid samples about 5 mL were homogenized before isolation and suspended in ratio of 1:10 in peptone buffered saline and incubated with shaking at 37ºC for 24–48h, then plating 100 µL of each agitated sample onto MacConkey's agar plates. Produced colonies were sub-cultured into nutrient agar slant and were subjected to various biochemical tests intended for conventional identification of *E. coli* [36].

### Detection of O-serogroups

The confirmed *E. coli* isolates were submitted to Center of food analysis, Faculty of Veterinary Medicine, Benha university, Egypt. The serotyping was performed using sets of *E. coli* antisera for rapid diagnosis (Denka Seiken Co., Japan) [37–39].

### Phenotypic identification of some virulence determinants of EHEC isolates

#### Biofilm assay using microtiter plate method

Formation of biofilm was determined quantitatively using a 96 well flat-bottomed polystyrene microtiter plate [40]. A pure colony from an overnight culture streaked on tryptic soy agar (TSA) (Sigma) was sub-cultured into 5 mL tryptic soy broth (TSB) (Sigma) supplemented with 0.25% anhydrous glucose for 24 h at 37 °C. Aliquots of 200 µL of pre-adjusted culture (OD_600_ = 0.25) were placed in four adjacent wells, and a negative control containing only TSB was included. After incubation of the plate at 37 °C without shaking for 18–24 h, the contents of the wells were aspirated and rinsed three times with 200µL PBS (pH = 7.4) to eliminate any non-adherent cells. The adherent cells were fixed using 150µL of absolute methanol for 15 min, then left to dry. The fixed biofilm was then stained with 150µL of 1% w/v crystal violet for 20 min. Following that, the plate was rinsed three times with distilled water and dried in the air. The stained biofilm was resolubilized by adding 150µL of (33% v/v) glacial acetic acid per well, and the OD of the tested isolates was determined at 490 nm using a microtiter plate reader. The mean of the four ODs for each isolate was calculated. The capacity of biofilm formation was assessed according to Schönborn et al., 2017 [40]. Briefly, cut-off optical density (ODc) was set as three standard deviations above the mean OD of the negative control. Strains were categorized as: non-biofilm producer, NBP (OD ≤ ODc); weak biofilm producer, WBP (ODc < OD_WBP_ ≤ 2 × ODc); moderate biofilm producer, MBP (2 × ODc < OD_MBP_ ≤ 4 × ODc) and strong biofilm producer, SBP (OD_SBP_ > 4 × ODc).

#### Swimming motility assay

Motility evaluation of EHEC isolates was performed according to Murinda et al., 2002, shields and Cathcart, 2012 [41, 42]. Shortly, the bacterial culture was tested for motility using triphenyltetrazolium chloride (TTC) media. After 24–48 h of incubation at 37 °C, growth was indicated by the appearance of red color. As motility occurred, red color was visible surrounding the inoculation area.

#### Hemagglutination assay

All isolates were tested for their ability to agglutinate human erythrocytes using slide agglutination technique [43] using human red blood cells (RBCs) (O-type) obtained from Blood Bank Gastroenterology Hospital, Mansoura university. Isolates were inoculated in nutrient broth media and incubated at 37°C for 48 h. Suspension of RBCs was prepared by washing of human RBCs (O-type) three times with physiological saline (0.9% NaCl) then, resuspended in saline to final conc of 3%. One drop of the bacterial culture was blended with one drop of the RBC suspension on a clean glass slide, and the mixture was vigorously stirred by a sterile tip to promote agglutination. The presence of erythrocyte clumping within 5 min of mixing was considered a positive agglutination result.

#### Screening of hemolysin activity

EHEC isolates' hemolytic activity was determined by streaking onto 5% blood agar plates. Plates were evaluated after incubation for 24 h at 37 °C [44]. Hemolysin production was determined quantitatively according to Rossignol et al., 2008 [45]. All EHEC isolates were cultured in TSB at 37 °C for 24 h with shaking and bacterial extract was obtained by centrifugation at 10,000 rpm for 10 min. A mixture of 500µL RBC suspension (2% O-type RBCs in 10 mM Tris HCl, pH 7.4) and 500µL bacterial extract previously prepared according to Rossignol was incubated for 2 h at 37 °C. A positive control (T) using 500µL 0.1% sodium dodecyl sulphate (SDS) and a negative control (B) using 500µL TSB with 500µL RBC suspension were included. After centrifugation at 10,000 rpm for 10 min, the amount of hemoglobin liberated in each sample (X) was measured at 540 nm. Three replicates were performed for each isolate. Hemolysis percentage was determined as follow:

$$\text{Hemolysis}\%= ((\text{X}-\text{B})/(\text{T}-\text{B})) \times 100$$

#### Serum resistance assay

Serum resistance of the strains was analyzed, as described by Vandekerchove et al., 2005 using a turbidimetric assay [46]. In a 96-well microtiter plate, 50 μL of bacterial culture (OD600 = 0.1) was combined with 150 μL of normal human serum obtained from Blood Bank Gastroenterology Hospital, Mansoura university. Using a microplate reader, the initial and final absorbance (after 3 h of incubation at 37 °C) were measured at 600 nm. The absorbance of each isolate was estimated by taking the average of three replicates. The percentage of remaining absorbance relative to the initial absorbance was calculated. The strain is classified as serum resistant (SR) if the remaining absorbance after 3 h was higher than 150%, intermediate resistant (IR) if it was between 125 and 150%, slow-intermediate resistant (S-I) if percent was between 100 and 125% and classified as serum sensitive (SS) if it was less than 100%.

### Molecular detection of some virulence genes

#### Genomic DNA extraction

The DNA templates were extracted from tested isolates by boiling method previously reported by Said et al., 2018 [47]. Concisely, purified *E. coli* colonies were suspended in 100 μL of sterile DNase free water in 0.2 mL sterile PCR tube and subjected to heat block in thermocycler at 95 °C for 10 min, followed by centrifugation at 10,000 rpm for 3 min and stored in −20°C. Supernatant served as DNA template for all the following PCR sets.

#### Screening of some toxin genes by PCR

Shiga toxin I *(stx1),* Shiga toxin II *(stx2),* intimin *(eae)* and hemolysin *(ehxA, ehlyA, hlyA, ehlyA* and *sheA)* genes were screened among the tested isolates using PCR. The reaction mixture of a total volume 25 μL consisted of 12.5 μL master mix (DreamTaq Green master), 2 μL of template DNA, 0.5µL from each primer listed in supplementary Table 2 (Invitrogen TM, UK) and the final volume completed with DNase free water.

#### *stx* gene subtyping

Subtyping of *stx1* and *stx2* genes was carried out as previously reported [48] using primer pairs listed in supplementary Table 2. Amplification was carried out using a DNA thermocycler with a predetermined cycling condition: initial denaturation at 94 °C for 5 min followed by 35 cycles each consisted of denaturation at 94 °C for 40 s, annealing at the temperature as stated in supplementary Table 2 and extension at 72 °C for 1 min, then final extension at 72 °C for 10 min. The amplified products were electrophoresed on 1.25% agarose gel stained with ethidium bromide and photographed under ultraviolet light (UV) light and compared with a DNA marker (Gene Ruler 100 bp, Thermo Fisher Scientific Tm, UK) to detect their sizes.

#### Clermont’s phylogenetic typing

Phylotyping of EHEC isolates was performed as previously described by Clermont et al., 2013 [6]. Amplicons were electrophoresed using 1.25% agarose gel stained with ethidium bromide and photographed under UV light. Phylotypes of EHEC isolates were assigned as: A, B1, B2, C, D, E, F, and Clade I.

#### Molecular typing by enterobacterial repetitive intergenic consensus PCR (ERIC-PCR)

Molecular genotyping of isolates was performed using (ERIC-PCR) using specific primers (supplementary Table 2) [49]. A total reaction of 25 µL consisted of 12.5µL of Dream Taq™ Green PCR Master Mix (2x), 0.5 µL of ERIC-1 (10 µM), 0.5 µL of ERIC-2 (10 µM), 8.5 µL of nuclease-free water and 3µL of template DNA. Amplification was conducted with the following conditions: initial denaturing at 94 °C for 5 min, 35 cycles of denaturation at 94 °C for 40 s annealing at 48 °C for 1 min, extension at 72 °C for 1.5 min, then the reaction was terminated by a final extension of 72 °C for 10 min. The amplified DNA fragments were separated on 2% agarose gel and compared with 100 bp plus DNA marker (Thermo Fisher Scientific™, UK) Cat. No. (SM0323). Following this, the gel was examined and evaluated using gelJ software. A similarity matrix was generated using Dice's coefficient and subsequently, the corresponding dendrogram was created utilizing the unweighted-pair group method with arithmetic averages (UPGMA) [50].

### Multi locus sequence typing (MLST)

#### Purification of PCR products

Amplification of MLST genes was performed using the previously described protocol [51] using the primers listed in (supplementary Table 2). PCR reaction was performed in a 100 μL reaction mixture containing 12 μL of the template DNA, 50 μL of Dream Taq™ Green PCR master mix (2X), each of the forward and reverse primer 2 μL and 34 μL of nuclease-free water. The standard cycling procedure was performed following the conditions: initial denaturation for 2 min at 95 °C, 35 cycles of denaturation at 95 °C for 1 min, annealing at variable temperature stated in (supplementary Table 2) for 30 s, extension at 72 °C for 2 min, and final extension at 72 °C for 7 min. The PCR products were analyzed by gel electrophoresis on 1.25% agarose gel stained with ethidium bromide and photographed under UV light, then the products with the target size were precisely cut from the gel and purified using Gene Jet Gel Extraction Kit (Thermo Scientific™).

#### DNA sequencing

The purified PCR products of the MLST were sequenced according to the protocol of applied biosystems big dye terminator v3.1 DNA sequencing reaction at Colors Medical Lab, Cairo, Egypt. Nucleotide sequences of the housekeeping genes were submitted to the *E. coli* MLST Database (https://pubmlst.org/bigsdb?db=pubmlst_escherichia_seqdef), and the EnteroBase Database (https://enterobase.warwick.ac.uk/species/ecoli/allele_st_search) to determine the sequence types and clonal complex.

#### Statistical analysis and data interpretation

The statistical analysis of the data was performed using GraphPad Prism (version 5.01), which involved the application of the chi-square test and Fisher's exact test, and Monte Carlo test. The significance of the obtained results was judged at *p*-value < 0.05.

## Results

### Isolation, identification, and serotyping of EHEC

Out of the 335 clinical, food and sewage water specimens collected, 105 (31%) isolates of *E. coli* were identified. The prevalence of the identified isolates among sources was as follows: 19/41 human urine, 46/77 human stool, 23/81 cheese, 2/6 yogurt, 2/2 raw milk, 7/27 cattle meat, 1/5 beef burger, 1/3 pastrami, 1/15chicken, 1/2 fish, 2/7 sewage water. No *E. coli* isolates were obtained from vegetables, sausage, or luncheon.

Serological typing classified the isolated *E. coli* into four pathotypes. They were distributed as 31 EHEC, 19 EPEC, 11 ETEC, 4 EIEC among clinical isolates and 21 EHEC, 9 EPEC, 8 ETEC, 2 EIEC among food and sewage water isolates. Thus, 52 (49.5%) of the identified isolates were assigned as EHEC which were subjected to further investigation throughout the study.

The prevalence of EHEC among *E. coli* isolated from urine and stool was 57.9% and 43.5%, respectively. Concerning food isolates, it was detected among 62.5% and 52.17% of *E. coli* isolated from meat and cheese, respectively. Also, all *E. coli* isolated from yogurt and sewage water were EHEC.

Among the detected 52 EHEC isolates, nine serotypes were identified. O111: H2 (23%) was the highest detected serotype followed by O91: H21 (21.2%) and O26: H11(19.2%). Moreover, other serotypes; O55: H7 (11.5%), O117:H4 (9.6%), O126: H21 (9.6%), O113: H4 (1.92%), O121: H7 (1.92%) and O103: H4 (1.92%); were detected. No significant difference was noticed between clinical, food and sewage water isolates concerning the distribution of serotypes (Table 1).

**Table 1:** Distribution of EHEC and serotypes among different sources

Source (Number of specimen)	No. of *E. coli* isolates	No. of EHEC isolates (%)	Serotypes (%)						
			O111: H2	O91: H21	O26: H11	O55: H7	O117: H4	O126: H21	Other Serotypes
**Urine (**41)	**19**	11 (57.9%)	2 (18.2%)	3 (27.3%)	1 (9.1%)	2 (18.2%)	2 (18.2%)	1 (9.1%)	-
**Stool (77)**	**46**	20 (43.5%)	3 (15%)	5 (25%)	1 (5%)	4 (20%)	2 (10%)	4 (20%)	1 (5%)
**Meat products (32)**	**8**	5 (62.5%)	3 (60%)	-	1 (20%)	-	-	-	1 (20%)
**Pastrami (3)**	**1**	-	-	-	-	-	-	-	-
**Cheese (81)**	**23**	12 (52.2%)	4 (33.3%)	1 (8.3%)	6 (50%)	-	-	-	1 (8.3%)
**Yogurt (6)**	**2**	2 (100%)	-	1 (50%)	1 (50%)	-	-	-	-
**Raw** **milk (2)**	**2**	-	-	-	-	-	-	-	-
**Chicken meat (15)**	**1**	-	-	-	-	-	-	-	-
**Fish (2)**	**1**	-	-	-	-	-	-	-	-
**Sewage water (7)**	**2**	2 (100%)	-	1 (50%)	-	-	1 (50%)	-	-
**Total number**	**105**	52 (49.50%)	12 (23%)	11 (21.20%)	10 (19.20%)	6 (11.50%)	5 (9.60%)	5 (9.60%)	3 (5.80%)

### Phenotypic identification of virulence determinants among EHEC isolates

#### Biofilm formation capacity

Biofilm formation capacity was observed in 51/52 (98%) of EHEC isolates. Concerning clinical isolates, 30/31 (96.77%) isolates formed biofilm. These isolates were classified as strong (1, 3.3% isolate), moderate (5, 16.66% isolates), weak (24, 80% isolates) – biofilm producers. All food and sewage water EHEC isolates were biofilm producers. They were classified equally into strong, moderate, and weak—biofilm producers (7, 33.3% isolates each) (Table 2).

**Table 2:** Distribution of virulence-associated phenotype detected among different EHEC isolates

Virulence factor	Number of Clinical isolates (%) (*n* = 31)	Number of food and sewage water isolates (%) (*n* = 21)	*P* value
Biofilm	30 (96.7%)	21 (100%)	0.405
-Strong producer	1(3.2%)	7 (33.33%)	0.003*
-Moderate producer	5 (16%)	7 (33.33%)	0.148
-Weak producer	24 (80%)	7 (33.33%)	0.001*
Motility	31(100%)	15 (71%)	0.001*
Blood hemolysis	0 (0%)	1 (4.7%)	0.219
Hemagglutination	14 (45%)	4 (19%)	0.052
Serum resistance	29 (93.5%)	19 (90.5%)	0.683
-Serum resistance	6 (20.69%)	7 (36.84%)	0.25
-Intermediate	5 (17.24%)	2 (10.53%)	0.494
-Slow intermediate	18 (62.07%)	10 (52.63%)	0.458

^*^: *P* value ≤ 0.05

Statistical analysis has revealed that strong biofilm formation capacity was significantly higher among EHEC from food and sewage water sources than from clinical isolates (*P* = 0.003) while weak biofilm formation capacity was significantly higher among clinical isolates (*P* = 0.001).

#### Phenotypic screening of motility, blood agglutination, hemolysin, and serum resistance

Regarding motility testing, 46/52 (88.5%) EHEC isolates showed growth diffusion with red color appeared around the stab line representing motility. While all clinical EHEC isolates were significantly motile, only 71% of food and sewage water EHEC isolates were motile (*P* = 0.001). Noticeably, our results revealed that all non-motile isolates were obtained from food sources.

For blood agglutination, we found that 18/52 (34.6%) isolates had the ability to agglutinate human RBCs within 5 min of vigorous stirring (Table 2). Blood agglutination was more observed among clinical isolates [14 (45%)] as compared to food and sewage water isolates [4 (19%)]. Hemolysis activity (β-hemolysis) was observed in only one yogurt isolate; the remaining 51 isolates (98%) were non-hemolytic (Table 2).

Among the 52 EHEC isolates, 48 (92.3%) isolates were serum resistant. 29 (93.5%) clinical isolates showed serum resistance [6 (20.69%) serum resistant, 5 (17.24%) intermediate, 18 (62.07%) slow intermediates. Serum resistance was detected in 19 (90.5%) of food and sewage water isolates [7 (36.84%) serum resistant, 2 (10.53%) intermediate, and 10 (52.63%) slow intermediates]. No significant difference was noticed between clinical, food and sewage water EHEC isolates regarding blood agglutination, hemolysis, and serum resistance (Table 2).

### Molecular detection of some virulence genes

#### Molecular detection of *stx1, stx2, eae* and hemolysin genes

All EHEC isolates were tested for toxin associated genes by PCR technique and were shown in (supplementary Fig. 1). Shiga toxin I (*stx1*) gene was harbored by only two clinical isolates. Subtyping of the two *stx1* positive isolates showed that both were of *stx*_*1a*_ subtype. No *stx*_*1c*_ or *stx*_*1d*_ subtypes were detected. Shiga toxin II (*stx2*) gene was more prevalent than *stx1* gene as it was detected among 39/52 (75%) isolates. *Stx2* positive clinical isolates (29/31,93.5%) were significantly more abundant than *stx2* positive food and sewage water isolates (10/21, 47.6%) (*P* = 0.001).

Regarding *stx* positive subtypes, three subtypes were obtained: *stx2b*, *stx2d* and *stx2g.* Among the 39 *stx2* positive isolates, *stx2g* was the highest detected subtype (17/39, 43.59%) followed by *stx2b* (8/39, 20.5%). *stx2d* was the least detected subtype (2/39, 5.13%)*.* None of the isolates were subtyped as *stx2a*, *stx2c*, *stx2e* or *stx2f.* Worth mentioning, five isolates harbored a combination of *stx2b* and *stx2g* subtypes simultaneously. Seventeen of *stx2* positive isolates could not be assigned to any of the tested subtypes. Significant difference in the prevalence of *stx2g* and un- subtyped isolates was found at (*P* = 0.019) and (*P* = 0.0008), respectively between isolates from different sources (Table 3).

**Table 3:** Prevalence of virulence genes among EHEC isolates from different sources

Virulence gene	Total number (%) (n = 52)	Number of EHEC isolates (%)		*P* value
		Clinical isolates (*n* = 31)	Food and sewage water isolates (*n* = 21)	
***stx1***	2 (3.8%)	2 (6.5%)	-	0.235
***Stx1* subtypes** ***stx1a***	2 (3.8%)	2(6.5%)	-	0.235
***stx2***	39 (75%)	29 (93.5%)	10 (47.6%)	0.001*
***stx2* subtypes**				
***stx2g***	17 (32.69%)	14 (45.16%)	3 (14.29%)	0.019*
***stx2b***	8 (15.38%)	6 (19.35%)	2 (9.52%)	0.335
***stx2d***	2 (3.8%)	2 (6.45%)	-	0.235
**Un-subtyped**	17 (32.69%)	12 (38.71%)	5 (23.8%)	0.0008*
***Eae***	19 (36%)	12 (38.7%)	7 (33.3%)	0.693
***sheA***	44 (84.6%)	28 (90.3%)	16 (76.2%)	0.165
***hlyA***	4 (7.7%)	4 (12.9%)	-	0.086

*EHEC* enterohaemorrhagic *E. coli*

*: *P *value ≤ 0.05

Additionally, *eae* gene was detected in 19/52 (36%) isolates. They were distributed as follows: 12/31 (39%) of clinical isolates and 7/21(33%) of food isolates, respectively. Ten out of 20 (50%) EHEC urine isolates and 2/11 (18%) EHEC stool isolates were *eae* positive. Regarding food isolates, we found that dairy products harbored *eae* gene more frequently than meat product where 6/14 (42.8%), 1/5 (20%) of dairy and meat products isolates, respectively were *eae* positive.

Hemolysin genes were detected among 47/52 (90%) EHEC isolates. Silent hemolysin (*sheA*) gene was detected in 44/52 (84.6%) isolates. The detection rate was higher among clinical isolates (28/31,90.3%) than food and sewage water isolates (16/21, 76.2%). While α hemolysin (*hlyA*) gene was harbored by only 4/52 (7.7%) isolates which were of clinical origin. Enterohemolysin-a (*ehlyA*) and enterohemolysin-x (*ehxA*) genes were not detected in any isolate (Table 3).

Virulence genes detected among EHEC isolates were represented in supplementary Fig. 1.

### Virulence genes profile

Gene profiles and their distribution among EHEC isolates were investigated. Fifteen different gene combinations of the tested virulence genes were obtained among the 52 EHEC (Table 4). The most prevalent profile was stx2, sheA (10/52,19.2%) followed by *stx2, stx2g, sheA, eae* (6/52, 11.5%) and *stx2, sheA, eae* and *stx2, stx2g, sheA* (5/52, 9.6% each). Nine profiles were unique as they were detected in only one isolate each. One meat product isolate and one sewage water isolate did not harbor any of the tested genes. Clinical isolates showed higher diversity as they revealed 14 different profiles, while only nine profiles were shown by food and sewage water isolates.

**Table 4:** Distribution of toxin gene profiles among EHEC isolated from different sources

Toxin genes profile	Total number of isolates (%) *n* = 52	Number of clinical isolates *n* = 31	Number of food and sewage water isolates *n* = 21
***stx2: stx2b, stx2g, eae, sheA, hlyA***	1 (1.9%)	1 (3.23%)	0
***stx1: stx1a, stx2: stx2g, eae, sheA***	1 (1.9%)	1 (3.23%)	0
***stx2: stx2b, stx2g, eae, sheA***	1 (1.9%)	1 (3.23%)	0
***stx1: stx1a, stx2: stx2g, stx2b***	1 (1.9%)	1 (3.23%)	0
***stx2: stx2b, eae, hlyA***	1 (1.9%)	1 (3.23%)	0
***stx2: stx2d, sheA, hlyA***	1 (1.9%)	1 (3.23%)	0
***stx2: stx2g, sheA, eae***	6 (11.5%)	3 (9.7%)	3 (14.28%)
***stx2: stx2g, stx2b, sheA***	2 (3.8%)	2 (6.45%)	0
***stx2, sheA, eae***	5 (9.6%)	4 (7.69%)	1 (4.76%)
***stx2: stx2b, sheA***	2 (3.8%)	0	2 (9.52%)
***stx2: stx2d, sheA***	1 (1.9%)	1 (3.23%)	0
***stx2: stx2g, sheA***	5 (9.6%)	5 (16.13%)	0
***stx2: stx2g***	1 (1.9%)	0	1 (4.76%)
***sheA, eae***	2 (3.8%)	1 (3.23%)	1 (4.76%)
***stx2, sheA***	10 (19.2%)	7 (22.58%)	3 (14.28%)
***stx2* alone**	1 (1.9%)	0	1 (4.76%)
***eae* alone**	2 (3.8%)	0	2 (9.52%)
***SheA* alone**	6 (11.5%)	0	6 (28.57%)
***HlyA* alone**	1 (1.9%)	1 (3.23%)	0
**No genes**	2 (3.8%)	0	2 (9.52%)

### Phylogenetic groups

Phylogenetic typing, based on Clermont’s typing, showed diversity among EHEC isolates. The tested isolates were assigned into seven phylogroups. B1 and C were the predominant phylogroups (15/52,28.8% each) followed by A and D (5/52, 9.6% each), B2 and E (3/52,5.77% each). Only two (3.8%) isolates were assigned to F phylogroup. The distribution of the detected phylogroups among EHEC isolates revealed no correlation between the phylotypes of isolates and their source *p* > 0.05 (Table 5).

**Table 5:** Phylogenetic distribution among clinical, food and sewage water EHEC isolates

Source	Phylogroup Number of isolates (%)							
	A	B1	B2	C	D	E	F	Unknown
**Clinical**	2 (3.84%)	7 (13.46%)	3 (5.76%)	6 (11.53%)	5 (9.61%)	3 (5.76%)	1 (1.92%)	4 (7.69%)
**Food and sewage water**	3 (5.76%)	8 (15.38%)	-	9 (17.31%)	-	-	1 (1.92%)	-
***P*** value	0.347	0.226	0.142	0.07	0.06	0.142	0.777	0.086

### Genotyping by using ERIC-PCR

The tested EHEC isolates were genotyped into 46 different patterns using ERIC-PCR. The largest pattern (P27) comprised four isolates that were obtained from food sources including cheese and meat samples.

**Fig. 1:**
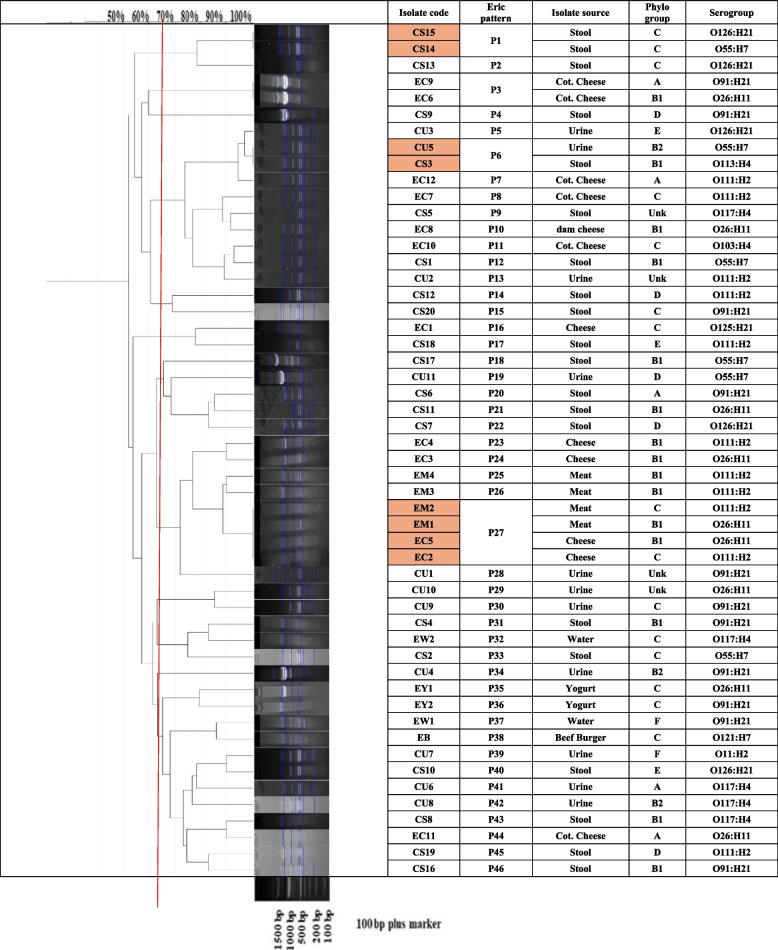
Molecular typing of EHEC isolates collected from various sources. Similarity of clustering analysis was performed using ERIC-PCR analysis. Dendrogram representing UPGMA and Dice coefficient for *E. coli*. CU: urine, CS: stool, EC: cheese, EM: meat, EW: sewage water, EY: yogurt and EB: beef burger isolates

The clonal relationship between EHEC isolates was analyzed using GelJ software and UPGMA clustering analysis (Fig. 1). Results revealed that the majority of isolates had shown similarity of > 70%. The obtained dendrogram clustered the isolates into sixteen clusters. In addition to a group of four (EM1, EM2, EC2 and EC5) and another pair (EC6 and EC9) of food isolates, two pairs of clinical isolates; (CS14 and CS15) and (CU5 and CS3); showed 100% similarity.

### Multiple sequence analysis of the seven house-keeping genes

The analysis of each gene among the three selected isolates is shown in supplementary Table 3.

#### Allele profiles, sequence typing and clonal complex of the selected EHEC isolates

The allelic number, the corresponding sequence type and clonal complex were designated by using MLST website. Our results had shown that the three isolates were assigned to different three sequence types (ST) depending on a specific combination of the allele numbers determined automatically using the Pubmlst website, where EC9 was assigned to ST120, CS9 was assigned to ST394 of clonal complex (CC) ST394 Cplx and CU11 was assigned to ST70 (supplementary Table 3).

#### Association between isolates, phylogroups, and phenotypic & genotypic characters

**Fig. 2:**
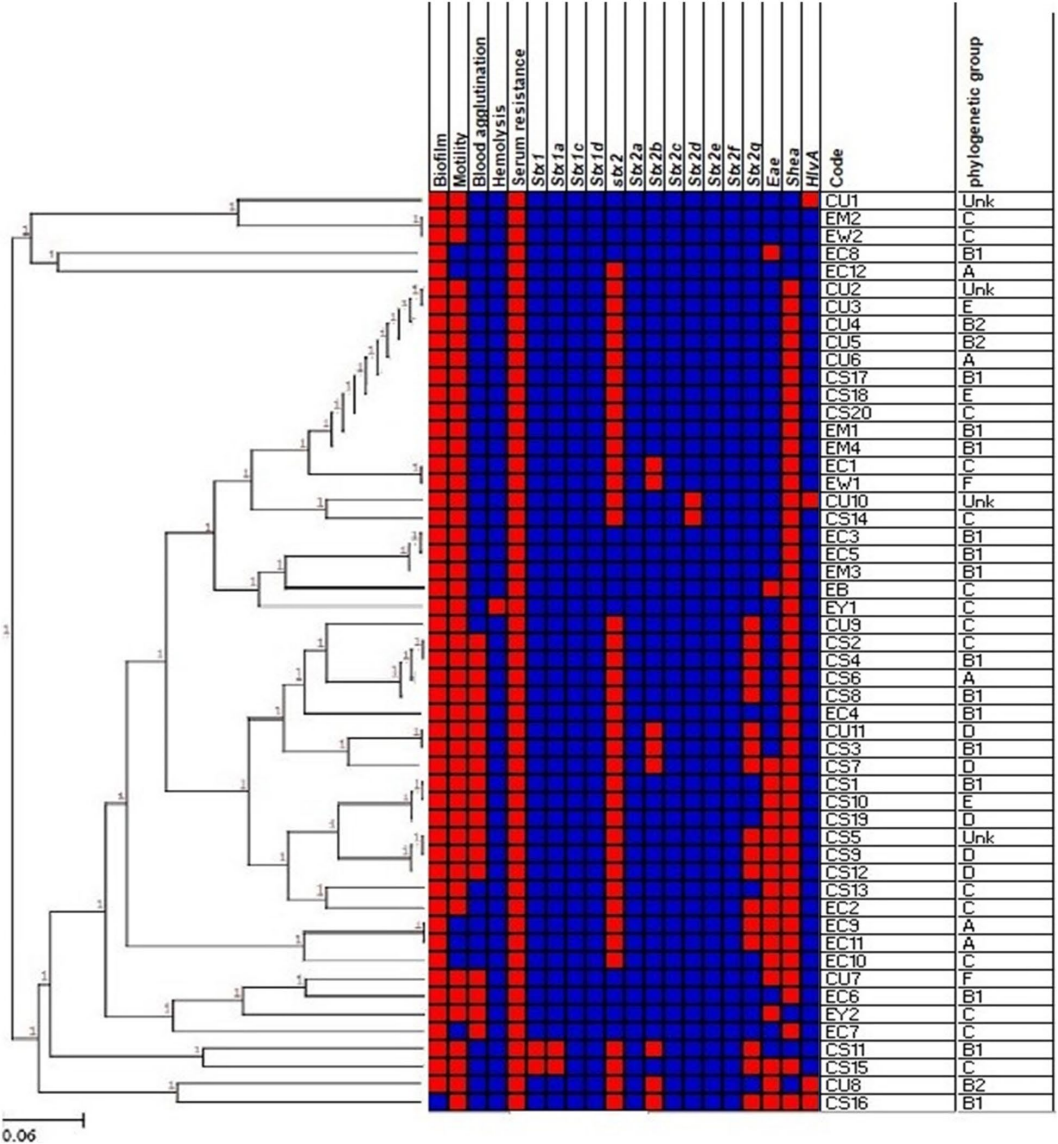
Heat map with hierarchical clustering of EHEC isolates collected from various sources based on their phenotypic and virulence genes reflecting differences between isolates. Blue represents negative, red represents positive result of phenotypic and genotypic characters**.** Hierarchical clustering was performed using UPGMA and Jaccard coefficient. CU: urine, CS: stool, EC: cheese, EM: meat, EW: sewage water, EY: yogurt and EB: beef burger isolates

Correlation matrix and hierarchical clustering with heat map (Fig. 2) were utilized to detect associations between the phenotypic & genotypic features and origin of the isolates to find any potential correlation between the isolates. Heat map classified isolates into two clusters comprising 20 patterns. Of our 52 EHEC isolates, 21 isolates showed 70% similarity.9 groups of isolates showed identical patterns in several traits that are: (EM2, EW2), (CU2, CU3, CU4, CU5, CU6, CS17, CS18, CS20, EM1 and EM4), (EC1, EW1), (EC3, EC5, EM3), (CS2, CS4, CS6, CS8), (CU11, CS3), (CS1, CS10, CS19), (CS5, CS9, CS12) and (EC9, EC11).

## Discussion

The emergence of O157 and non-O157 EHEC foodborne pathogens has become a major concern worldwide [52]. They cause serious disease outbreaks and severe illness in humans such as diarrhea, HC and potentially fatal HUS [53]. The great attention to non-O157 EHEC could be related to the increased prevalence of these organisms in human and animal infections, as well as increased public awareness of the danger of infection caused by non-O157 serogroups [54]. Children under the age of five are more vulnerable and at danger of dying from diarrhea caused by *E. coli* infection [55].

The goal of this study was to explore the prevalence of EHEC among different sources in Egypt and investigate their molecular characteristics. In addition, the distribution of virulence factors among these isolates was also investigated. A total of 52 (49.5%) EHEC isolates had been identified (Table 1). The prevalence of EHEC was similar in both clinical (31/65, 47.7%), food and sewage water (21/40, 52.5%) isolates, where none of these isolates was O157. On the other hand, Ahmed et al. (2017) reported that 33% of their EHEC isolates was O157 [56]. In Germany, one-third of non-O157 HUS-causing EHEC was associated with O26, O103, O111, and O145 serogroups [14, 57].

Serogroups O26, O111, O103, and O145 accounted for one-third of human infections other than O157:H7 such as diarrhea and HUS, in Germany and caused global outbreaks [17, 58]. Of these serogroups, O26, O103 and O111 were detected in our study. Similarly, Ahmed et al. (2017) reported the presence of these three serogroups among their EHEC isolates.

A high prevalence of EHEC was observed among our urine and stool isolates (Table 1), where prevalence in urine was greater than that of stool. Some studies reported variable rates of isolation of EHEC from clinical sources in Iran (30.77%) [59] and South Africa (0.4%) [60].

Meat products are a substantial cause of human EHEC infections [61]. Rahimi, et al. 2012 reported the presence of EHEC in meat products in Netherland (10.4%), England (13.4%), and Iran (8.2%) [62]. Similarly, EHEC was detected in 14.3% of all meat samples in our study. Interestingly, researchers detected similar rates of EHEC in animal products at 9.5%, [63, 64]. None of our vegetable samples harbored EHEC, despite the detection of EHEC in vegetables from different countries [65, 66].

Raw milk and raw-milk products have been implicated in many diseases and even fatalities [67]. Several countries reported the detection of variable levels of *stx* gene in their milk products, which is the most crucial virulence for EHEC-associated infections [68]. It was found that 7 out of 12 EHEC cheese isolates were *stx* gene positive. This finding is alarming and necessitates strict control over the cheese industry to prevent the spread of this organism. Thankfully, yogurt samples that were identified as EHEC among our isolates did not harbor *stx* gene.

*E. coli* is known to persist in natural environments due to the formation of biofilm [69]. The majority (98.1%) of our non-O157 EHEC isolates were biofilm producers, which is consistent with several previous studies [19, 70]. Strong biofilm production was observed among 3.2% of clinical isolates, where 9% of urine isolates were strong biofilm producers (Table 2). A recent study in Egypt, reported that 22.7% of urine isolates were strong biofilm producers [71].

Many bacterial pathogens rely on flagellar motility in the early stages of infection. While Sherfi, et al. 2013 concluded that E. coli motility and indole production are related, only 88.5% of our indole-positive isolates were motile [72].

Hemagglutination (HA) of erythrocytes is believed to be a major virulence in *E. coli* strains that cause extraintestinal illnesses in human [73]. A higher detection rate of HA was found among clinical isolates (45%) than in food isolates (19%). HA was observed at 60% from our fecal isolates while at 18% from our urine isolates (Table 2). It was reported that HA was observed at 25% from urine isolates while at 4% from fecal isolates [43].

Prolonged presence of hemolytic *E. coli* strains in the host could potentially lead to the onset of extra-intestinal infections [74, 75], bloodstream infection and sepsis [76]. Beta-hemolytic activity was detected in a yogurt isolate and not detected in clinical isolates (Table 2). However, previous studies observed higher percent of hemolytic activity including 42.2%, 25%, 16.8% in *E. coli* isolates for [71, 75] and [77], respectively.

Serum resistance characteristic enables *E. coli* to evade the complement system and increases risk of developing septic shock and mortality [78, 79]. In this study, 92.3% of EHEC isolates showed varying levels of serum resistance (Table 2).

The ability to harbor *stx* gene is a trait shared by EHEC/STEC isolates. In our research, *stx2* gene (75%) was more prevalent than *stx1* (3.8%) and a percent of 3.8% of EHEC isolates harbored both genes (Table 3). This result is supported by Sallam et al.,2013 in that *stx1* and *stx2* genes were found in the isolated EHEC strains in 46.7% and 86.7%, respectively [80] and by Jajarmi et al., 2017 who reported that *stx1* (52%), *stx2* (64%) and 16% of EHEC isolates that harbored both genes were detected among their isolates [81]*.*The *stx1* was found among isolated STEC at low rates 0.16%, 5.2% as reported by Kargar M, Homayoon M. 2015 and Tarazi*, *et al. 0.2021, respectively [82] and [83].

It has been reported that STEC strains that produce *stx2* gene are more likely to cause HUS and could cause more severe neurological symptoms in piglets than strains producing just *stx1* or both *stx1* and *stx2*, whereas *stx1*-producing strains induce only diarrhea without systemic complications [84].

Subtyping of the *stx1*^+^ isolates showed that they were of *stx*_*1a*_ subtype. The same finding was reported by Elsayed et al who found that no *stx1c* was detected in non-o157 *E. coli* isolates [35].

The *stx2* group is composed of *stx2a, stx2b, stx2c, stx2d, stx2e, stx2f, and stx2g* subtypes [48]. Our results indicated that *stx2g* (43.6%) was the most prevalent subtype among *stx2* positive isolates followed by *stx2b* (20.5%) then *stx2d* (5.1%), while *stx2a, stx2c, stx2e and stx2f* were not detected among our isolates (Table 3).

Elsayed et al. , 2021 [35] demonstrated that no *stx2a*, *stx2d*, *stx2f*, or s*tx2g* subtypes were detected in their isolates. In contrast, Jajarmi et al., identified *stx1a* (52%), *stx2a* (44%), *stx2c* (44%), and *stx2d* (30%) in selected STEC isolates [81]. Supporting our findings, *stx2c* have been detected often in isolates from HUS patients, whereas *stx2d* is often isolated from cases of uncomplicated diarrhea [85]. The *stx2e* subtype is predominantly isolated from pigs and pork products [86, 87]. Schmidt et al., 2000 came to a conclusion that *stx2f* appears to be closely related to STEC of bird and pigeons origins [88].

The *eae* gene is one of the most common pathogenic genes found in the environment [89, 90] that makes STEC strains more virulent for humans. However, eae-lacking STEC strains caused a minority of sporadic cases of HUS [91]. Several putative non intimin based adhesion has been described, such as long polar fimbriae and STEC auto-agglutinating adhesin in non-O157:H7 STEC [92, 93]. In our study *eae* gene was detected in 19/52 (36%) isolates.

The hemolysin gene that is one of the key pathogenic components of *E. coli* was found in 92% of our EHEC isolates and its subtypes were detected. Of which, silent hemolysin gene *(sheA)* was the most prevalent one. Alpha- hemolysin (*hlyA*) gene was detected in 7.7% of clinical isolates only (Table 3). Similarly, low prevalence of *hlyA* gene was reported in 2.25%, 0.9% of cheese and raw milk, respectively [94].

Fifteen different combinations of virulence genes were detected among our 52 EHEC isolates**.** The most prevalent profile is *stx2, sheA* (19.2%) followed by *stx2, stx2g, sheA, eae* (11.5%), *stx2, sheA, eae* (9.6%) and *stx2, stx2g, sheA* (9.6%) (Table 4). Eklund M, et al*.*2002 reported 11 combinations in Finland [95]. Furthermore, we detected *stx* gene subtypes combinations among clinical isolates only, where four isolates harbored (*stx2b* with *stx2g)* while *(stx1a* with *stx2g)* and (*stx1a* with *stx2b* and *stx2g)* were harbored each by only one isolate, which is similar to Elsayed M, et al.2021 [35]. Various combinations were previously detected [96–98]. Supporting our findings Dong, et al.2017 reported that *stx1d*, *stx2e, and stx2f* were not detected among *E. coli* isolates [99].

A multiplex PCR system was carried out to classify *E. coli* strains into phylogenetic groups. Most of our EHEC isolates belonged to non-pathogenic B1 and C phylogroups, and few strains belonged to group E, F (Table 5) which is supported by Rúgeles et al*.*, 2010 [100]. In our EHEC isolates 8/52 belonged to pathogenic B2 and D phylogroups and they were of clinical origin and all harbored *stx2* and hemolysin genes while only five of them harbored *eae* gene.

The ERIC-PCR was applied to examine the genetic similarity of EHEC isolates obtained from diverse sources. The isolates were genotyped into forty-six different patterns using ERIC-PCR. Similarity of 100% was observed between many isolates which may be indicative of similar origin of dissemination of similar isolates (Fig. 1). Opposing our finding that there was no correlation between ERIC patterns and serotypes as O55:H7 isolates were highly diverged and found in 6 different groups (P1, P6, P12, P18, P19, and P33), Dalla costa et al. reported that related clones like O55:H7 and O157:H7 displayed similar ribotypes and clustered together in a dendrogram [30]. Furthermore, Nicholas Waters et al. demonstrated the relationship between whole-genome phylogeny and the phylotypic classification and concluded that phylotypes and classification at the whole-genome are correlated [101]. On the other hand, our study advocated that there was no correlation between phylogeny and ERIC clusters.

The MLST technique is used to study genetic relatedness of isolates, and closely related species can be categorized into clonal complexes [33]. The selection of isolates for MLST was based on using two isolates from clinical source, both belonged to pathogenic phylogroup (D) with one isolate from food source that belonged to non-pathogenic phylogroup (A). The three selected EHEC isolates were classified into three different sequence types demonstrating a clonal complex diversity among the isolates. The EC9 isolated from cheese was assigned to endemic clone ST120 belonging to commensal phylogenetic group A. This endemic clone of ST120 has been previously reported in China [102]. This necessitates immediate action to prevent the spread of this clone. The ST120 strains identified from cormorants were CTX-M-15 producers and belonged to commensal phylogenetic group B1 [103]. Regarding CS9 isolate from stool, it belonged to pathogenic D phylogroup and ST394 (clonal complex: ST394 Cplx). Zahra et al., 2018 detected ST394 from sewage around Pakistan [104].

Alarmingly, ST394 has been associated with sporadic diarrheal outbreaks in various countries [105]. The ST394 that was previously isolated from raw milk belonging to phylogroup D was confirmed to be CTX-M15-producing [106].

Urine isolate (CU11) in our study was assigned to epidemic clone ST70 and belonged to pathogenic D phylogroup. Alarmingly, ST70 was previously detected in the acute intensive care unit, Venezuela indicating that its ability to induce frequent outbreaks [107].

The three detected STs were previously characterized as emerging ESBLs that are capable of degrading expanded-spectrum cephalosporins and monobactams [108, 109] and resistant to aminoglycosides and fluoroquinolones [110, 111] as well as resistance to gentamicin and ciprofloxacin [108] leaving just a few reliable alternative therapies [112, 113].

By comparing results of the three selected isolates by two different typing techniques; ERIC typing method that classified them into 3 variable patterns (P3, P4 and P19) and MLST that classified them into three different sequence types (ST70, ST120, ST394) demonstrating a genetic diversity among the isolates and ensures accuracy of both techniques.

Associations between phenotypic, genotypic features and serotypes of isolates (Fig. 2) revealed that CU4, CU5 isolates that showed similarity in several traits were obtained from urine and both belonging to pathogenic phylogroup B2 and CS9, CS12 isolates that were obtained from stool showed similarity in several traits are belonging to pathogenic phylogroup D.

In conclusion, our study advocated that cheese and meat products represent dangerous threats as source of EHEC infections. Various serotypes were detected among clinical, food and sewage water isolates including: O111: H2, O91: H21, O26: H11, O55: H7, O117: H4, O126: H21, O113: H4, O121: H7 and O103: H4 and this represents a potential risk for public health. It is worrying that 58% of our cheese isolates harbored *stx2* gene that is the most important virulence trait for EHEC infections along with 77% of total EHEC isolates having “*stx2*, hemolysin and or *eae*” gene combination making them highly virulent. The MLST technique was applied, ST394 detected has been associated with acute and chronic sporadic diarrheal outbreaks in both developed and developing countries, detected epidemic clone ST70 was found to be capable of inducing frequent outbreaks. The endemic ST120 detected in this work was isolated from cheese necessitating critical action to prevent spread of this clone and it is our duty to emphasize the importance of strict control over cheese factories, since they pose the threat of infection with hazardous microorganisms. Our study advocated that pathogenic phylogroups B2 and D were found solely within clinical isolates. With high rate of *stx2* gene detection in clinical isolates, representing a major threat for the dissemination of EHEC among hospitals and community-acquired *E. coli* isolates. Alarmingly, clinical EHEC isolates harbored high virulence genes score and combinations that is causing a public health problem. Our findings might have a substantial influence on the development of preventive strategies for *E. coli* infections through the identification of potential sources that could serve as a vehicle for the transmission of these pathogenic bacteria.

## Supplementary Information

Supplementary Material 1.

Supplementary Material 2.

Supplementary Material 3.

Supplementary Material 4.

Supplementary Material 5.

## Data Availability

All data generated or analyzed during this study are included in this published article [and its supplementary information files]. The datasets generated and/or analyzed during the current study are available at Sequence Read Archive (SRA) and accession numbers are provided in Table 6.

**Table 6:** Accession numbers of sequenced genes for Multi locus sequence typing (MLST) of three EHEC isolates

Isolate Code	Gene	SRA Accession	Biosample Accession	BioProject Accession	Sample name
CU11	*adk*	SRR25293914	SAMN36465545	PRJNA995194	ACU11
CU11	*fumC*	SRR25304273	SAMN36496207	PRJNA995194	FCU11
CU11	*gyrp*	SRR25304275	SAMN36496220	PRJNA995194	GCU11
CU11	*icd*	SRR25304289	SAMN36496233	PRJNA995194	ICU11
CU11	*mdh*	SRR25304302	SAMN36496247	PRJNA995194	MCU11
CU11	*purA*	SRR25304315	SAMN36496313	PRJNA995194	PCU11
CU11	*recA*	SRR25304395	SAMN36496324	PRJNA995194	RCU11
CS9	*adk*	SRR25302836	SAMN36493589	PRJNA995194	ACS9
CS9	*fumC*	SRR25302936	SAMN36493621	PRJNA995194	FCS9
CS9	*gyrp*	SRR25302915	SAMN36493619	PRJNA995194	GCS9
CS9	*icd*	SRR25302913	SAMN36493618	PRJNA995194	ICS9
CS9	*mdh*	SRR25302979	SAMN36493954	PRJNA995194	MCS9
CS9	*purA*	SRR25303012	SAMN36494089	PRJNA995194	PCS9
CS9	*recA*	SRR25303002	SAMN36494071	PRJNA995346	RCS9
EC9	*adk*	SRR25298601	SAMN36468095	PRJNA995346	AEC9
EC9	*fumC*	SRR25302531	SAMN36471198	PRJNA995346	FEC9
EC9	*gyrp*	SRR25302530	SAMN36471199	PRJNA995346	GEC9
EC9	*icd*	SRR25302529	SAMN36471200	PRJNA995346	IEC9
EC9	*mdh*	SRR25302528	SAMN36471201	PRJNA995346	MEC9
EC9	*purA*	SRR25302527	SAMN36471202	PRJNA995346	PEC9
EC9	*recA*	SRR25302526	SAMN36471203	PRJNA995346	REC9 Accession numbers of sequenced genes for Multi locus sequence typing (MLST) of three EHEC isolates
