# Supplementary material for: Characterization of non-O157 enterohemorrhagic Escherichia coli isolated from different sources in Egypt
Source: BMC Microbiol. 2024 Nov 21;24:488. doi: 10.1186/s12866-024-03636-3 (PMC11580514; doi:10.1186/s12866-024-03636-3)
Supplement: Supplementary file 1 — Supplementary Material 1. [file 12866_2024_3636_MOESM1_ESM.docx]

**Supplementary table 1: Distribution of the sources of the different specimens collected in the study.**

| **Isolate**  **ID** | **Source** | **Location** | | **Isolate**  **ID** | **Source** | **Location** | |
| --- | --- | --- | --- | --- | --- | --- | --- |
| **M1** | Meat | Butcher shops at in Mansoura city | B1 | **C30- C36** | Cheese | Local supermarkets at Mansoura and Damietta cities | S30- S36 |
| **M2- M9** | Meat |  | B2- B9 | **C37, C38** | Cheese |  | S37, S38 |
| **M10- M12** | Meat |  | B10- B12 | **C39** | Cheese |  | S39 |
| **M13- M15** | Meat |  | B13- B15 | **C40- C54** | Cheese |  | S40- S54 |
| **M16- M19** | Meat |  | B16- B17 | **C55- C62** | Cheese |  | S55- S62 |
| **M20- M24** | Meat |  | B20- B24 | **C63- C74** | Cheese |  | S63- S74 |
| **M25- M27** | Meat |  | B25- B27 | **C75- C81** | Cheese |  | S75- S81 |
| **M28- M38** | Luncheon | Local supermarkets in Mansoura and Damietta cities | S88- S98 | **Y1, Y2** | Yogurt |  | S82- S83 |
| **M39- M43** | Luncheon |  | S99- S103 | **Y3, Y4** | Yogurt |  | S84- S85 |
| **M44- M50** | Luncheon |  | S104- S110 | **Y5, Y6** | Yogurt |  | S86 |
| **M51- M60** | Luncheon |  | S111- S120 | **ML1, ML2** | Milk |  | S87 |
| **M61- M71** | Luncheon |  | S120- S130 | **Ch1- Ch4** | Cooked chicken | Chicken shop at Mansoura city | C.S1- C.S4 |
| **M72- M75** | Luncheon |  | S130- S133 | **Ch5- Ch7** | Cooked chicken |  | C.S5- C.S7 |
| **M76** | Luncheon |  | S134 | **R1** | Roasted chicken | Local supermarkets at Mansoura city | S152 |
| **M77- M79** | Beef burger |  | S135- S137 | **R2, R6** | Roasted chicken |  | S153- S157 |
| **M80, M81** | Beef burger |  | S138- S139 | **R7, R8** | Roasted chicken |  | S158, S159 |
| **M82- M84** | Ground beef |  | S140- S142 | **N1** | Lemon | Greengrocers | G.G1 |
| **M85, M86** | Ground beef |  | S143- S144 | **T** | Tomato |  | G.G2 |
| **M87- M89** | Pastrami |  | S145- S147 | **P1** | Pepper |  | G.G3 |
| **M90, M91** | Sausage |  | S148- S149 | **P2- P6** | Pepper |  | G.G4- G.G8 |
| **M92, M93** | Sausage |  | S150- S151 | **P7, P8** | Pepper |  | G.G9, G.G10 |
| **C1- C5** | Cheese |  | S1- S5 | **S.W1- S.W3** | Sewage water | Sewer from different locations at Damietta (1-3) and Mansoura (4-7) cities | SE1- SE3 |
| **C6- C11** | Cheese |  | S6- S11 | **S.W4- S.W7** | Sewage water |  | SE4- S7 |
| **C12- C29** | Cheese |  | S12- S29 | **F1, F2** | Fish | Fish shop in Mansoura city | F.S1, F.S2 |

**B1-B27**: butcher shops; **S1-S159:** local supermarkets; **C.S1**- **C.S7:** chicken shop; **G.G:** greengrocers; **F.S:** fish shop.
