# Supplementary material for: Characterization of non-O157 enterohemorrhagic Escherichia coli isolated from different sources in Egypt
Source: BMC Microbiol. 2024 Nov 21;24:488. doi: 10.1186/s12866-024-03636-3 (PMC11580514; doi:10.1186/s12866-024-03636-3)
Supplement: Supplementary file 2 — Supplementary Material 2. [file 12866_2024_3636_MOESM2_ESM.docx]

Supplementary table 2: List of primers used for this study.

| Virulence traits | Virulence gene | Sequence | Amplicon size  bp | Annealing temp C° | Reference |
| --- | --- | --- | --- | --- | --- |
| Shiga toxin I | ***Stx1-F*** | TCCTGGTACAACTGCGGTTAC | 505 | 58 | [[46](#_ENREF_46)] |
|  | ***Stx1-R*** | ACGCACTCTTCCATCTACCG |  |  |  |
| Shiga toxin II | ***Stx2-F*** | CTGGCGTTAATGGAGTTCAGTGG | 381 | 53 | [[47](#_ENREF_47)] |
|  | ***Stx2-R*** | CCTGTCGCCAGTTATCTGACA |  |  |  |
| Intimin | ***Eae-F*** | GACCCGGCACAAGCATAAGC | 384 | 62 | [[48](#_ENREF_48)] |
|  | ***Eae-R*** | CCACCTGCAGCAACAAGAGG |  |  |  |
| Hemolysin genes  EHEC hemolysin | ***ehxA F*** | TCTGTATCTGCGGGAGTTAG | 136 | 54 | [[49](#_ENREF_49)] |
|  | ***ehxA R*** | CAACGTGCTCAAACATAGCC |  |  |  |
| [enterohemolysin](http://allie.dbcls.jp/pair/ehlyA;enterohaemolysin.html) | ***ehlyA F*** | TCGCAATCACATCACAACC | 810 | 54 |  |
|  | ***ehlyA R*** | CCAGCAGTTCGTCATCATCTGAA |  |  |  |
| Hemolysin A | ***hlyA F*** | GTCTGCAAAGCAATCCGCTGCAAATAAA | 561 | 54 |  |
|  | ***hlyA R*** | CTGTGTCCACGAGTTGGTTGATTAG |  |  |  |
| Silent hemolysin | ***sheA F*** | GAGGCGAATGATTATGACTG | 920 | 54 |  |
|  | ***sheA R*** | ACTTCAGGTACCTCAAAGAG |  |  |  |
| Stx1 subtyping  Shiga toxin 1a | ***stx1a-F*** | CCTTTCCAGGTACAACAGCGGTT | 478 | 58 | [[42](#_ENREF_42)] |
|  | ***stx1a-R*** | GGAAACTCATCAGATGCCATTCTGG |  |  |  |
| Shiga toxin 1c | ***stx1c-F*** | CCTTTCCTGGTACAACTGCGGTT | 252 | 58 |  |
|  | ***stx1c-R*** | CAAGTGTTGTACGAAATCCCCTCTGA |  |  |  |
| Shiga toxin 1d | ***stx1d-F*** | CAGTTAATGCGATTGCTAAGGAGTTTACC | 203 | 58 |  |
|  | ***stx1d-R*** | CTCTTCCTCTGGTTCTAACCCCATGATA |  |  |  |
| Stx2 subtyping  Shiga toxin 2a | ***stx2a-F2*** | GCGATACTGRGBACTGTGGCC | 349 | 54 |  |
|  | ***stx2a-R3*** | CCGKCAACCTTCACTGTAAATGTG |  |  |  |
| Shiga toxin 2b | ***stx2b-F1*** | AAATATGAAGAAGATATTTGTAGCGGC | 251 | 54 |  |
|  | ***stx2b-R1*** | CAGCAAATCCTGAACCTGACG |  |  |  |
| Shiga toxin 2c | ***stx2c-F1*** | GAAAGTCACAGTTTTTATATACAACGGGTA | 177 | 54 |  |
|  | ***stx2c-R2*** | CCGGCCACYTTTACTGTGAATGTA |  |  |  |
| Shiga toxin 2d | ***stx2d-F1*** | AAARTCACAGTCTTTATATACAACGGGTG |  | 54 |  |
|  | ***stx2d-R1*** | TTYCCGGCCACTTTTACTGTG | 179 |  |  |
|  | ***stx2d-O55-R*** | TCAACCGAGCACTTTGCAGTAG | 235 |  |  |
|  | ***stx2d-R2*** | GCCTGATGCACAGGTACTGGAC | 280 |  |  |
| Shiga toxin 2e | ***stx2e-F1*** | CGGAGTATCGGGGAGAGGC | 411 | 54 |  |
|  | ***stx2e-R2*** | CTTCCTGACACCTTCACAGTAAAGGT |  |  |  |
| Shiga toxin 2f | ***stx2f-F1*** | TGGGCGTCATTCACTGGTTG | 424 | 54 |  |
|  | ***stx2f-R1*** | TAATGGCCGCCCTGTCTCC |  |  |  |
| Shiga toxin 2g | ***stx2g-F1*** | CACCGGGTAGTTATATTTCTGTGGATATC | 573 | 54 |  |
|  | ***stx2g-R1*** | GATGGCAATTCAGAATAACCGCT |  |  |  |
| Quadruplex phylotyping ChuA | ***chuA.1bF*** | ATGGTACCGGACGAACCA | 288 | 59 | [[5](#_ENREF_5)] |
|  | ***chuA.2bR*** | TGCCGCCAGTACCAAAGACA |  |  | [[50](#_ENREF_50)] |
| yjaA | ***yjaA.1bF*** | CAAACGTGAAGTGTCAGGAG | 211 | 59 | [[5](#_ENREF_5)] |
|  | ***yjaA.2bR*** | AATGCGTTCCTCAACCTGTG |  |  |  |
| TspE4.C2 | ***TspE4C2.1bF*** | CACTATTCGTAAGGTCATCC | 152 | 59 |  |
|  | ***TspE4C2.2bR*** | AGTTTATCGCTGCGGGTCGC |  |  |  |
| arpA | ***AceK.F*** | AACGCTATTCGCCAGCTTGC | 400 | 59 | [[51](#_ENREF_51)] |
|  | ***ArpA1.R*** | TCTCCCCATACCGTACGCTA |  |  |  |
| Group E (arpA) | ***ArpAgpE.F*** | GATTCCATCTTGTCAAAATATGCC | 301 | 57 | [[52](#_ENREF_52)] |
|  | ***ArpAgpE.R*** | GAAAAGAAAAAGAATTCCCAAGAG |  |  |  |
| Group C (trpA) | ***trpAgpC.1F*** | AGTTTTATGCCCAGTGCGAG | 219 | 57 |  |
|  | ***trpAgpC.2R*** | TCTGCGCCGGTCACGCCC |  |  |  |
| Internal control (trpA) for group C and E | ***TrpBA.F*** | CGGCGATAAAGACATCTTCAC | 489 | 59 | [[53](#_ENREF_53)] |
|  | ***TrpBA.R*** | GCAACGCGGCCTGGCGGAAG |  |  |  |
| ERIC- PCR | ***ERIC-1*** | ATGTAAGCTCCTGGGGATTCAC | Variable | 48 | [[24](#_ENREF_24)] |
|  | ***ERIC-2*** | AGTAAGTGACTGGGGTGAGCG |  |  |  |
| Adenylate Kinase | ***Adk. F*** | ATTCTGCTTGGCGCTCCGGG | 583 | 54 | [[45](#_ENREF_45)] |
|  | ***Adk. R*** | CCGTCAACTTTCGCGTATTT |  |  |  |
| Fumarate  Hydratase | ***FumC. F*** | TCACAGGTCGCCAGCGCTTC | 806 | 54 |  |
|  | ***FumC. R*** | GTACGCAGCGAAAAAGATTC |  |  |  |
| Dna Gyrase | ***GyrB. F*** | TCGGCGACACGGATGACGGC | 911 | 60 |  |
|  | ***GyrB. R*** | ATCAGGCCTTCACGCGCATC |  |  |  |
| Isocitrate/iso-propyl malate  Dehydrogenase | ***Icd. F*** | ATGGAAAGTAAAGTAGTTCCGGCACA | 878 | 54 |  |
|  | ***Icd. R*** | GGACGCAGCAGGATCTGTT |  |  |  |
| Malate-dehydrogenase | ***Mdh. F*** | AGCGCGTTCTGTTCAAATGC | 799 | 60 |  |
|  | ***Mdh. R*** | CAGGTTCAGAACTCTCTCTGT |  |  |  |
| Adenyl succinate Dehydrogenase | ***PurA. F*** | CGCGCTGATGAAAGAGATGA | 816 | 54 |  |
|  | ***PurA. R*** | CATACGGTAAGCCACGCAGA |  |  |  |
| ATP/GTP  BINDING MOTIF | ***RecA. F*** | CGCATTCGCTTTACCCTGACC | 780 | 58 |  |
|  | ***RecA. R*** | TCGTCGAAATCTACGGACCGGA |  |  |  |

**bp:** base pair ***F*:** forward ***R*:** reverse
