# Supplementary material for: Characterization of non-O157 enterohemorrhagic Escherichia coli isolated from different sources in Egypt
Source: BMC Microbiol. 2024 Nov 21;24:488. doi: 10.1186/s12866-024-03636-3 (PMC11580514; doi:10.1186/s12866-024-03636-3)
Supplement: Supplementary file 3 — Supplementary Material 3. [file 12866_2024_3636_MOESM3_ESM.docx]

Supplementary table 3: Allele profiles of EHEC isolates according to Acthman scheme.

1. **Isolate EC9 profile**

| **Isolate EC9** | | | | | | | | | |
| --- | --- | --- | --- | --- | --- | --- | --- | --- | --- |
| **Locus** | ***adk*** | ***fumC*** | ***gyrB*** | ***icd*** | ***mdh*** | ***purA*** | ***recA*** | **ST** | **CC** |
| **Allele** | **49** | **4** | **44** | **9** | **11** | **35** | **7** | **ST120** |  |
| **Length of**  **locus (bp)** | **536** | **469** | **460** | **518** | **452** | **478** | **510** |  |  |

1. **Isolate CS9 profile**

| **Isolate CS9** | | | | | | | | | |
| --- | --- | --- | --- | --- | --- | --- | --- | --- | --- |
| **Locus** | ***adk*** | ***fumC*** | ***gyrB*** | ***icd*** | ***mdh*** | ***purA*** | ***recA*** | **ST** | **CC** |
| **Allele** | **21** | **35** | **61** | **52** | **5** | **5** | **4** | **ST394** | **ST394**  **Cplx** |
| **Length of**  **locus (bp)** | **536** | **469** | **460** | **518** | **452** | **478** | **510** |  |  |

1. **Isolate CU11 profile**

| **Isolate CU11** | | | | | | | | | |
| --- | --- | --- | --- | --- | --- | --- | --- | --- | --- |
| **Locus** | ***adk*** | ***fumC*** | ***gyrB*** | ***icd*** | ***mdh*** | ***purA*** | ***recA*** | **ST** | **CC** |
| **Allele** | **34** | **36** | **28** | **25** | **28** | **16** | **4** | **ST70** |  |
| **Length of**  **locus (bp)** | **536** | **469** | **460** | **518** | **452** | **478** | **510** |  |  |

**ST:** Sequence type**; CC:** Clonal complex
