# Supplementary material for: Characterization of non-O157 enterohemorrhagic Escherichia coli isolated from different sources in Egypt
Source: BMC Microbiol. 2024 Nov 21;24:488. doi: 10.1186/s12866-024-03636-3 (PMC11580514; doi:10.1186/s12866-024-03636-3)
Supplement: Supplementary file 4 — Supplementary Material 4. [file 12866_2024_3636_MOESM4_ESM.docx]

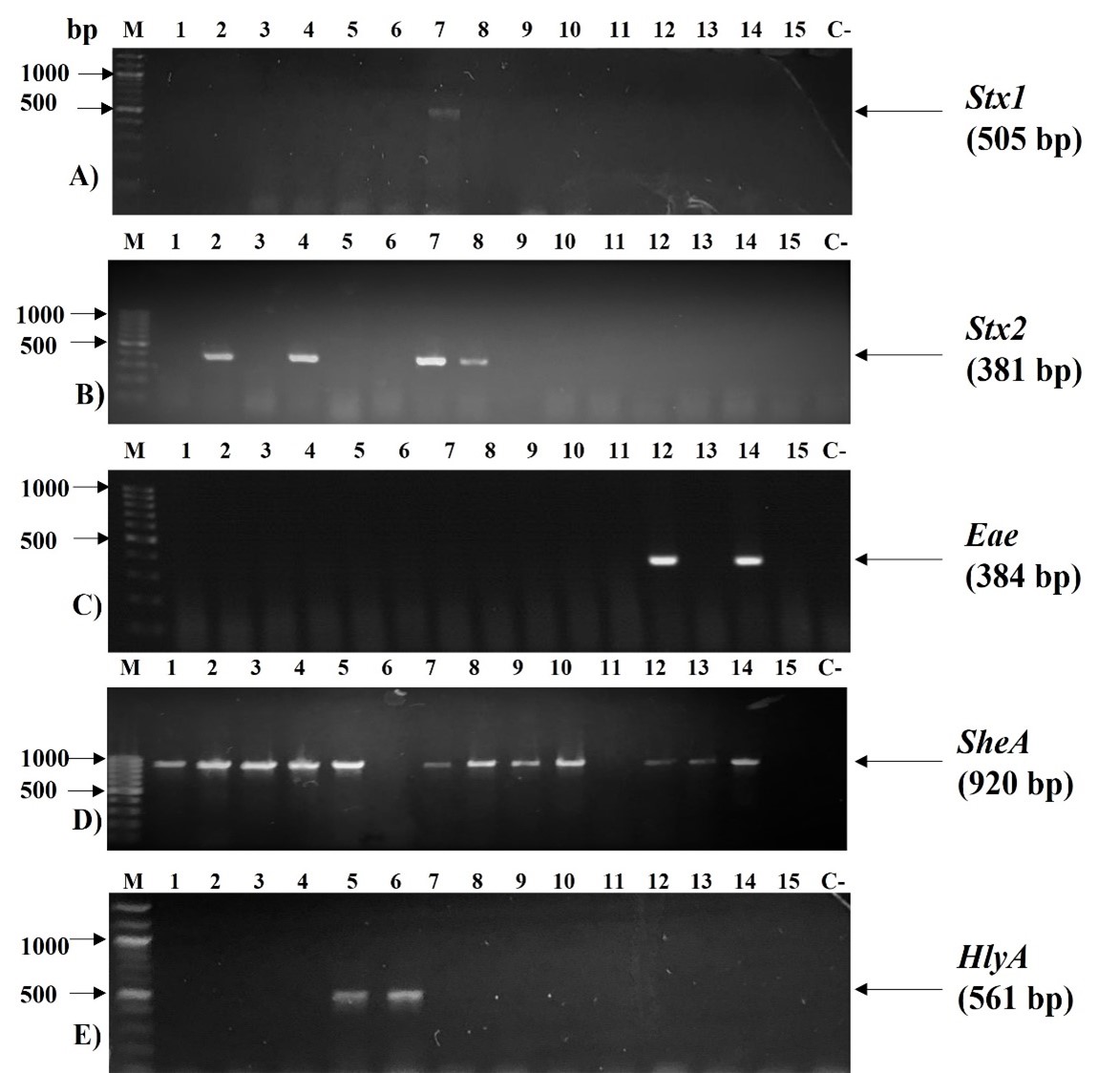


Figure S1: Agarose gel electrophoresis of EHEC-specific gene amplicons identified by PCR. Lane M: 100 bp DNA ladder; lane C-: negative control, Lanes 1-15 are representative PCR runs for amplified DNA samples indicating positive amplicon genes of: (A): stx1, (B): stx2, (C): eae, (D): sheA, (E): hlyA, bp: base pair.

M 1 2 3 4 5 6 7 8 9 10 11 12 13 -C


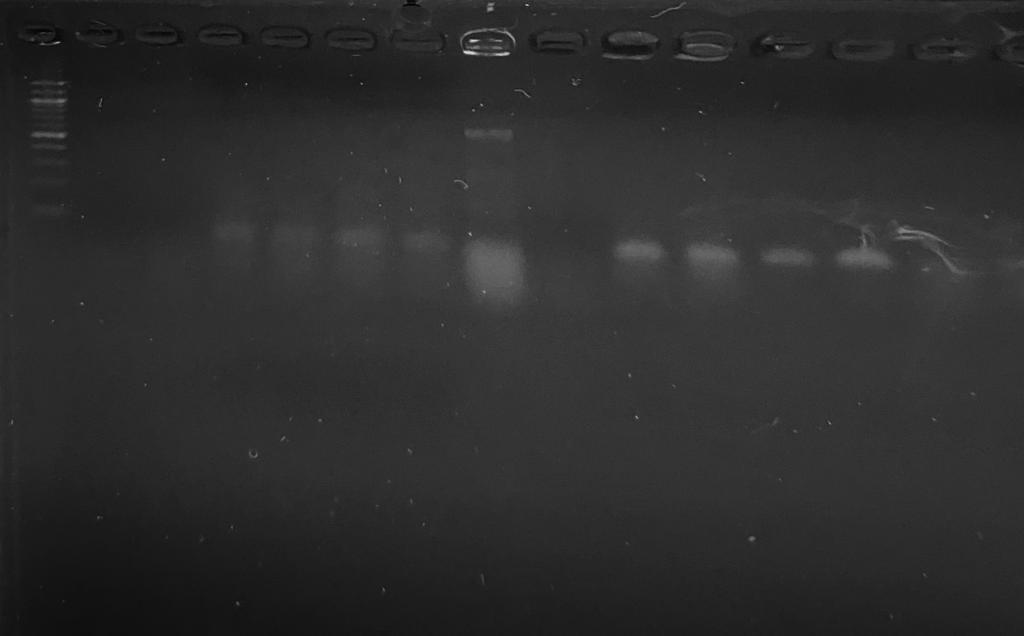


**1000 bp**

**500 bp**

Figure S1 A: Agarose gel electrophoresis of PCR for detection of *stx1* gene in *E. coli*isolates. Lane M: 100 bp DNA ladder; lane C-: negative control, bp: base pair. Lane 7: positive sample with band of amplicon size 505 bp. Lanes 1-6, 8-13: negative samples.

M 1 2 3 4 5 6 7 8 9 10 11 12 13 14 15 -C


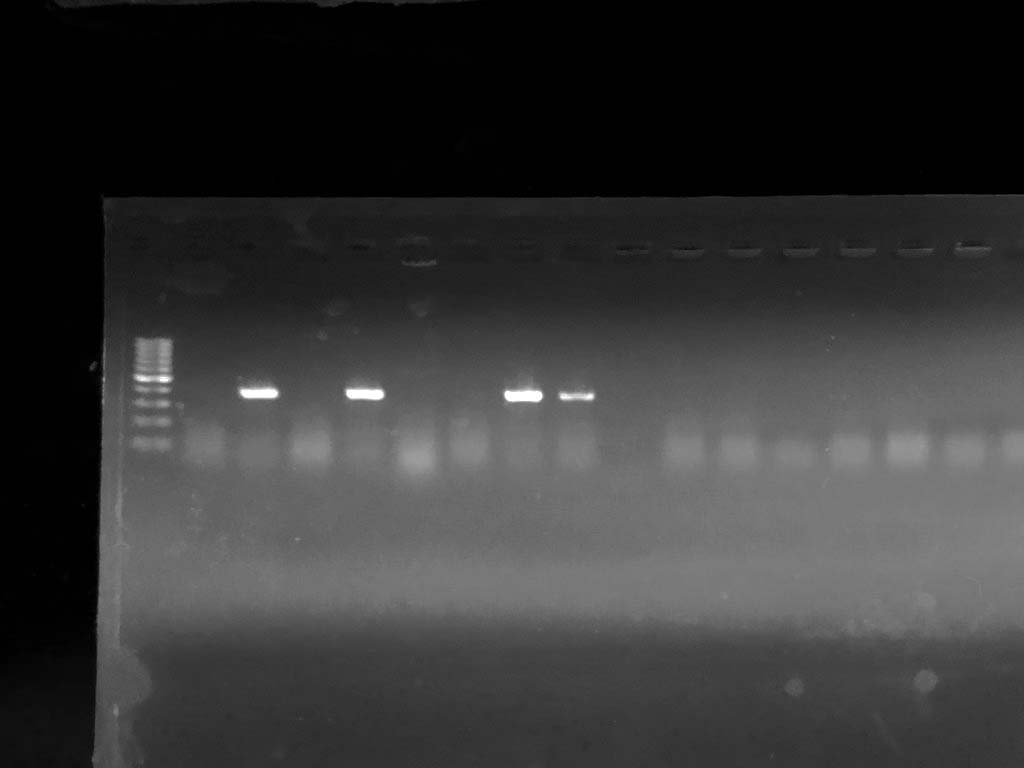


**500 bp**

**1000 bp**

Figure S1 B: Agarose gel electrophoresis of PCR for detection of *stx2*gene in*E. coli*isolates*.* Lane M: 100 bp DNA ladder; lane C-: negative control, bp: base pair. Lanes 2,4,7,8: positive samples with band of amplicon size 381bp. lanes 1,3,5,6, 9-15: negative samples.

M 1 2 3 4 5 6 7 8 9 10 11 12 13 14 15 -C


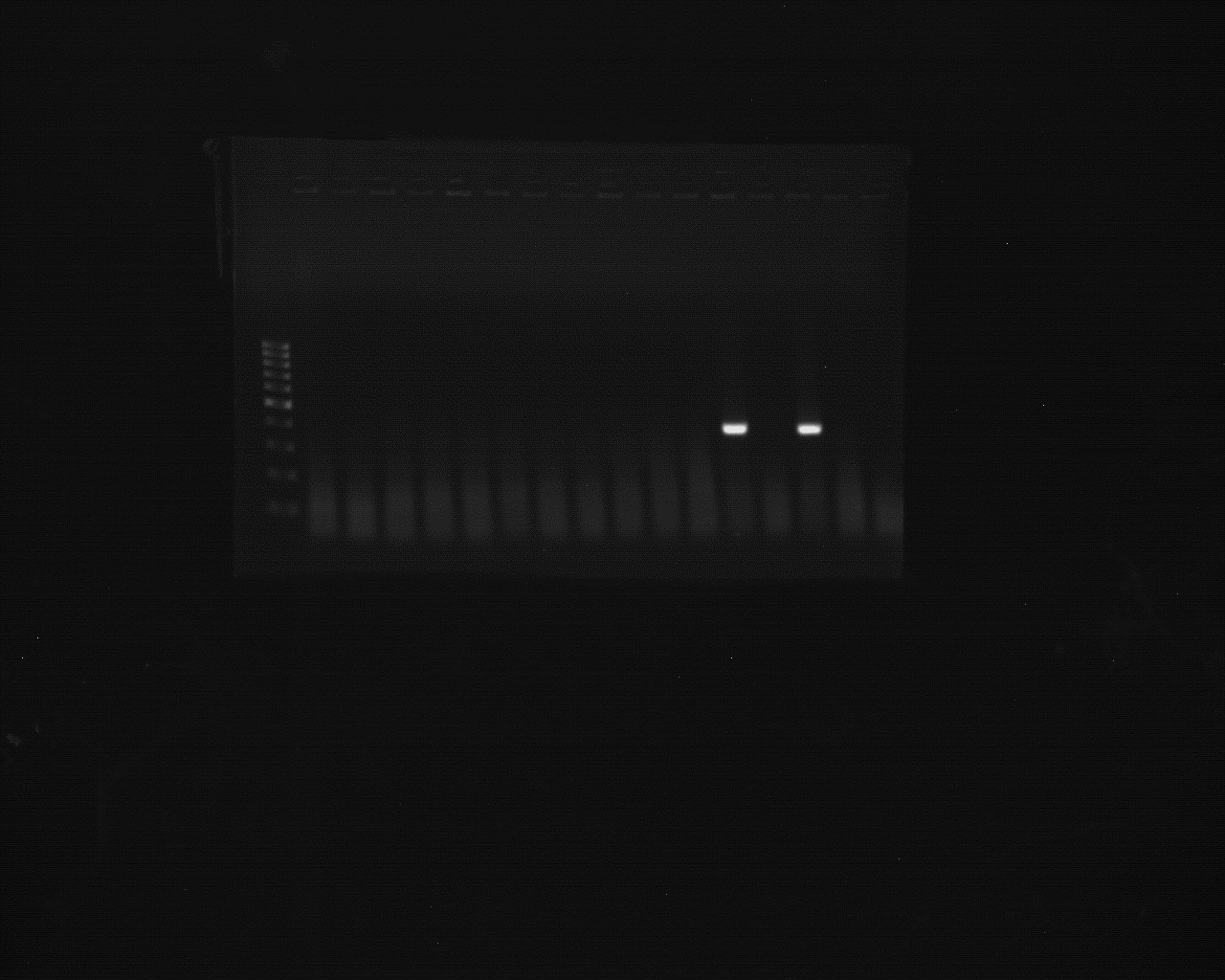


**500 bp**

**1000 bp**

Figure S1 C: Agarose gel electrophoresis of PCR for detection of *eaeA* gene in*E. coli* isolates. Lane M: 100 bp DNA ladder; lane C-: negative control. Lanes 12,14: positive samples with band of amplicon size 384 bp. Lanes 1-11,13,15: negative samples.

M 1 2 3 4 5 6 7 8 9 10 11 12 13 14 15 -C


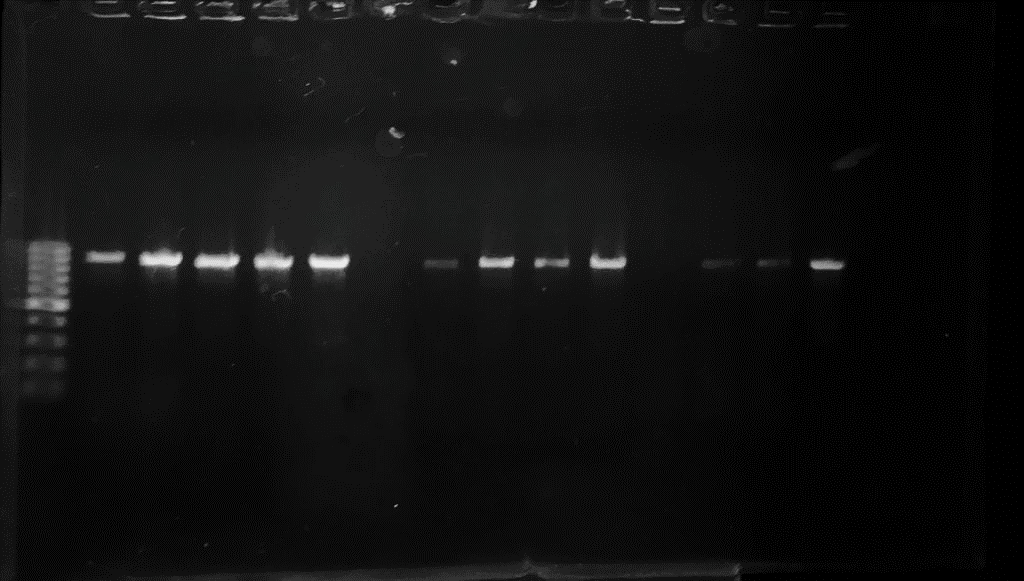


**500 bp**

**1000 bp**

Figure S1 D: Agarose gel electrophoresis of PCR for detection of *sheA* gene in*E. coli* isolates. Lane M: 100 bp DNA ladder; lane C-: negative control. Lanes 1-5, 7-10, 12-14: positive samples with band of amplicon size 920 bp. lanes 6,11,15 negative samples.

M 1 2 3 4 5 6 7 8 9 10 11 12 13 14 15 -C


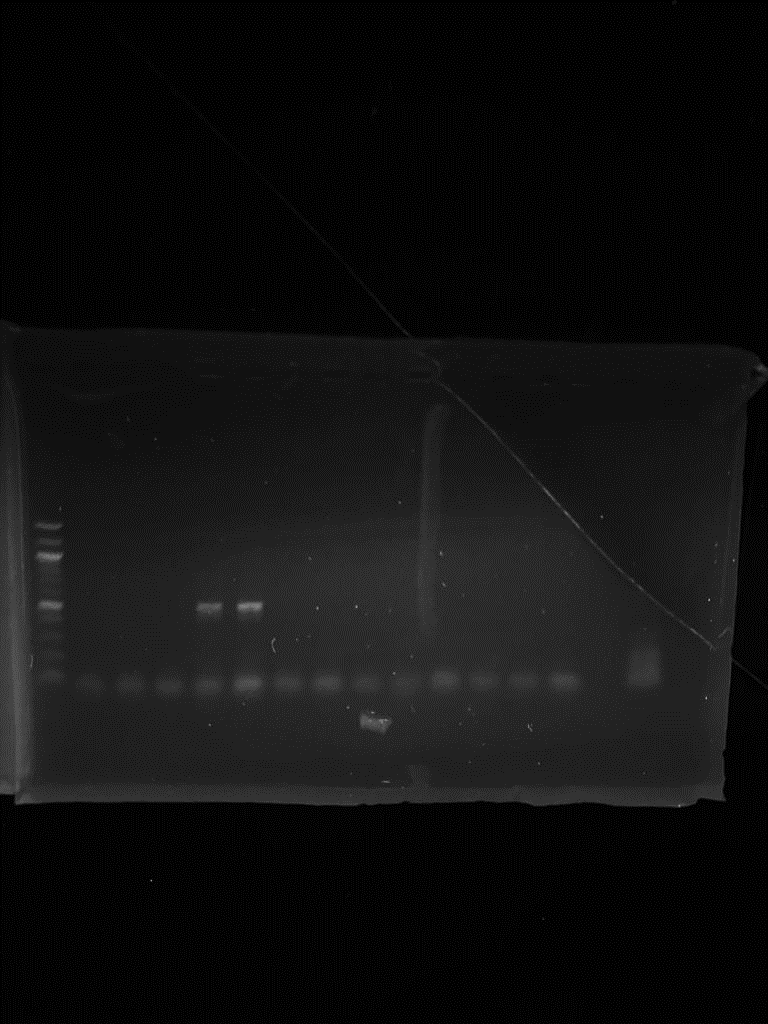


**1000 bp**

**500 bp**

Figure S1 E: Agarose gel electrophoresis of PCR for detection of *hlyA*  gene in*E. coli* isolates. Lane M: DNA ladder; lane C-: negative control. Lane 4,5: positive samples with band of amplicon size 561bp. Lanes 1-3, 7-15: negative samples.
