# Supplementary figures and images for: Characterization of non-O157 enterohemorrhagic Escherichia coli isolated from different sources in Egypt
Source: BMC Microbiol. 2024 Nov 21;24:488. doi: 10.1186/s12866-024-03636-3 (PMC11580514; doi:10.1186/s12866-024-03636-3)

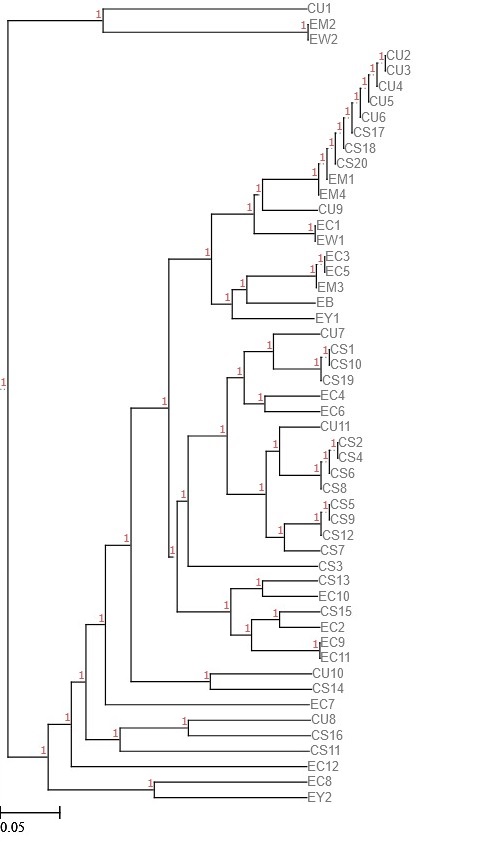


Figure S2: Dendrogram used in heat map.

Supplement: Supplementary file 5 — Supplementary Material 5. [file 12866_2024_3636_MOESM5_ESM.docx]
